# Pathological and molecular study of kidneys in apparently healthy cattle and sheep with special reference to *Leptospira* species in central and northern Jordan

**DOI:** 10.14202/vetworld.2023.2488-2496

**Published:** 2023-12-25

**Authors:** Nabil Q. Hailat, Zaid A. Mafrag, Mohammad H. Gharaibeh, Ibrahim M. Alzuheir

**Affiliations:** 1 Department of Veterinary Pathology and Public Health Faculty of Veterinary Medicine, Jordan University of Science and Technology, P.O. Box 3030 Irbid, 22110 Jordan; 2 Department of Basic Veterinary Medical Science, Faculty of Veterinary Medicine, Jordan University of Science and Technology, P.O. Box 3030 Irbid, 22110 Jordan; 3 Department of Veterinary Medicine, Faculty of Agriculture and Veterinary Medicine, An-Najah National University, P.O. Box 7 Nablus, Palestine

**Keywords:** cattle, Jordan, leptospirosis, polymerase chain reaction, renal lesions, sequencing, sheep

## Abstract

**Background and Aim:**

Renal pathological conditions can cause significant economic losses for livestock owners. This study investigated and described the gross pathology and histopathology of lesions found in the kidneys of sheep and cattle, with particular attention to the presence of *Leptospira* spp.

**Materials and Methods:**

Three hundred and sixty kidneys from apparently healthy sheep and cattle (208 and 152, respectively) were collected from slaughterhouses in Jordan for gross and histopathological examinations, multiplex polymerase chain reaction (PCR) analysis, and gene sequencing of *Leptospira* spp.

**Results:**

Histopathological analysis of the samples revealed the following conditions: interstitial nephritis (4.44%), glomerulonephritis (4.16%), acute tubular necrosis (17.22%), and renal congestion (4.72%). In addition, in 26.9% of the samples, hyaline material was observed in the interstitium of the cortex and medulla. The PCR results revealed that 83 (23.1%) of the 360 samples were positive for *Leptospira* spp. 16S rRNA, 42/152 (28%) of cattle, and 41/208 (20%) of sheep. Four samples (two sheep and two cattle) were sequenced and deposited in GenBank with accession numbers from OL701310 to OL701313. Basic Local Alignment Search Tool search and nucleotide similarities between OL701311 and OL701313 resulted in the highest similarities with different *Leptospira borgpetersenii* strains, whereas OL701310 showed the highest nucleotide similarity (99.2%) with the *Leptospira interrogans* strain. Similarly, phylogenetic analysis revealed that OL701311 to OL701313 clustered together with different serovars of *L. borgpetersenii*, whereas OL701310 clustered with the *L. interrogans* clade.

**Conclusion:**

This is the first study to reveal a close association between pathogenic *Leptospira* spp. and kidney disorders in Jordanian cattle and sheep. These findings may help expand the current understanding of the causes and mechanisms of renal disease in cattle and sheep and contribute to developing more effective prevention and treatment programs.

## Introduction

Leptospirosis is an infectious disease that affects both humans and animals. The disease is caused by *Leptospira* spp., which is a spirochete bacterium [1–3]. In humans, *Leptospira* infections attack the kidneys and liver, which can lead to morbidity and mortality. In animals, mainly sheep and cattle, after a variable incubation period, *Leptospir*a circulate, infect, and replicate in different organs such as kidneys, liver, lungs, genital tracts and central nervous system and results in abortion and stillbirth. In addition, *Leptospira* causes chronic infections in the kidneys, uterus, and udder, which can reduce milk production [2, 4]. However, in most cases, the infection was subclinical. Leptospirosis is generally transmitted by direct contact with the body fluids of infected animals or indirectly through contact with contaminated pasture or water [5]. Kidneys are paired organs that serve multiple functions, including the filtration of waste and toxic products; regulation of acid-base balance; urine excretion; hormonal secretion, such as renin regulating blood pressure; and regulation of water and salt concentrations [6]. The kidneys consist of complex basic structural and functional units, which are well-interconnected components that receive 25% of blood circulation [7]. Renal lesions are identified according to their morphological components of the kidney and are traditionally classified as those affecting the glomeruli, renal tubules, and interstitium. Some infectious agents can target the functional units of the kidneys, causing chronic renal lesions and, in most cases, leading to renal failure and “end-stage renal disease” [8].

Renal diseases cause significant economic losses every year due to the high death rate, the condemnation of affected organs and carcasses, loss of body weight, and cost of medication and veterinary services. While some renal diseases are cured, others progress to chronic and end-stage renal disease [9, 10]. Renal diseases are not rare in animals but are often undetected until they are at an advanced stage. Hence, information collected from abattoir studies remains an ideal source for the evaluation and control of renal diseases in animals [11].

The aim of this study were to (i) investigate and describe the gross pathology and histopathology of lesions found in the kidneys of apparently healthy sheep and cattle slaughtered in the central and northern parts of Jordan; (ii) estimate the prevalence of *Leptospira* spp. in the kidney tissues of cattle and sheep using polymerase chain reaction (PCR); and (iii) conduct DNA sequencing to compare the sequences obtained with those of *Leptospira* spp. deposited in a worldwide database.

## Materials and Methods

### Ethical approval

The study protocol was approved by the Jordan University of Science and Technology (JUST) Animal Care and Use Committee (ACUC, Project # 368/2020).

### Study period and location

This cross-sectional study was conducted from September 2020 to February 2021. The sampling area included slaughterhouses in the main cities in Central and Northern Jordan, namely, Amman (n = 122), Irbid (n = 202), Al-Rumtha (n = 27), and Al-Mafraq (n = 9). The samples were processed at Pathology Laboratory at the Faculty of Veterinary Medicine at JUST.

### Sample collection

Three hundred and sixty kidneys from each imported and local cattle and sheep (208 from sheep and 152 from cattle) were collected randomly after gross examination. Tissue samples were aseptically collected and divided into two parts. The first part was kept in a sterile stomacher bag, whereas the second part was stored in 10% buffered formalin immediately after slaughter and transported in an icebox for further testing to the Pathology Laboratory at the Faculty of Veterinary Medicine at JUST.

### Histopathological examination

Renal tissue samples in buffered formalin were processed for routine embedding in paraffin wax, as previously described by Ajayi *et al*. [12]. For each sample, 4–5-mm-thick sections were cut, deparaffinized, stained with hematoxylin and eosin, and examined by a light microscope (Nikon, Japan). Each slide was examined for approximately 10 min, after which pathological findings were recorded and representative photos were by a digital camera (Nikon, Japan).

### DNA extraction, PCR amplification, and gene sequencing

Total DNA was extracted from the kidney samples using the Wizard Genomic DNA Purification Kit (Qiagen, Germany). *Leptospira* 16S ribosomal RNA in the samples was amplified by nested PCR using two primer sets, as described previously by Esteves *et al*. [13]: forward-A 5′-GGCGGCGCGTCTTAAACATG-3′ and reverse -B 5′-TTCCCCCCATTGAGCAAGATT-3′ for the first PCR; and 5′-TGCAAGTCAAGCGGAGTAGC-3′ and nested -B 5′-TTCTTAACTGCTGCCTCCCG-3′ for the nested PCR. Nuclease-free water was used as a negative control. Polymerase chain reaction was initiated with an initial denaturation at 95°C for 5 min, followed by 35 cycles of amplification comprising denaturation (94°C for 30 s), annealing (58°C for 30 s), primer extension (72°C for 1 min), and a final extension of 7 min at 72°C. The amplified fragments were confirmed on a 1.5% agarose gel by ethidium bromide staining under ultraviolet light. Polymerase chain reaction products with the expected sizes (292 bp) were sent for Sanger sequencing to Macrogen (Seoul, South Korea) using forward primers. Sequence quality was initially verified using Finch TV 1.4 software (https://digitalworldbiology.com/FinchTV). The Basic Local Alignment Search Tool (BLAST) of the National Center for Biotechnology (NCBI) was used to determine nucleotide similarities and the most closely related *Leptospira* spp. (http://blast.ncbi.nlm.nih.gov/) [14]. Good-quality sequences were submitted to GenBank. The genome sequences and reference *Leptospira* spp. strains were aligned using the ClustalW package of the MEGA X software (https://www. megasoftware.net/) [15, 16]. Phylogenetic trees were generated using the neighbor-joining method with 1000 bootstrap replicates.

## Results

### Gross and histopathological findings

**Figure-1:**
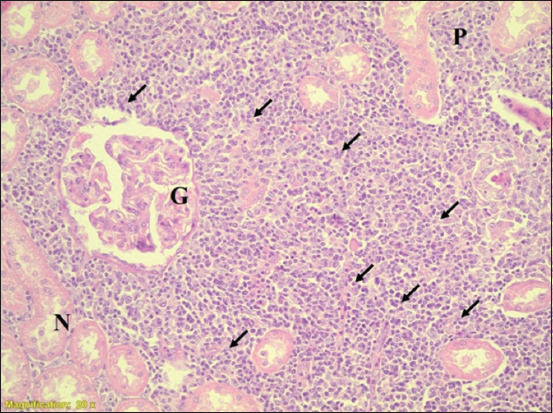
Section of kidney cortex of calves. Interstitial nephritis (arrows). G=Glomerulus, P=Proximal convoluted tubule, infiltration of leukocyte (arrows), N=Necrosis of tubular cells. (H&E) 20×.

**Figure-2:**
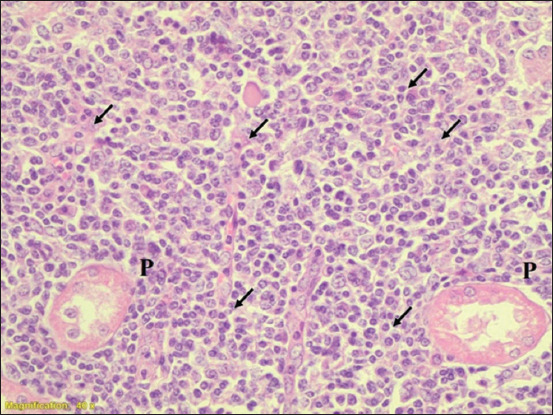
Section of kidney cortex of calves. P=Proximal convoluted tubule, infiltration of leukocyte (arrows), (H&E) 40×.

**Figure-3:**
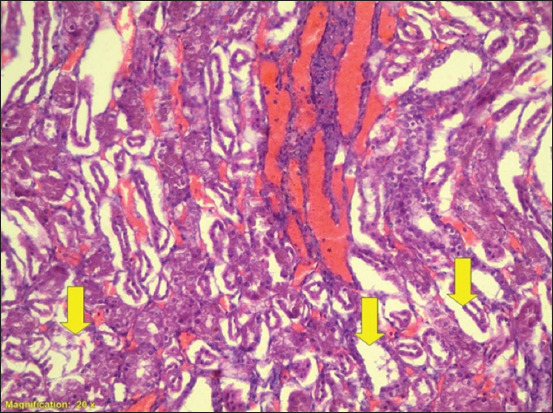
Section of kidney medulla of calves. Acute tubular necrosis. Kidney tubules (yellow arrows). (H&E) 20×.

Table-1 shows that the severity of lesions with acute tubular necrosis (ATN) or interstitial nephritis (IN) were graded as +, ++, +++, or ++++. Grades ++ and +++ had higher percentiles than grades + and ++++ for leptospirosis in the cattle kidney tissue samples, whereas grades + and ++ had the highest percentiles in the sheep kidney tissue samples. For both species, grade ++ was the highest among the four categories (39% and 45%), whereas grade ++++ was the lowest (8% and 4%) in cattle and sheep, respectively). The PCR results were also higher in grade ++ compared to the other grades (10% and 13%). Because grade ++ has a large number of samples, moderate lesion and higher percentile in PCR results, it was compared with grade ++++ which has very severe lesion and low percentile in PCR results. When grade ++ was compared with grade ++++, the prevalence of *Leptospira* spp. was 5-fold in cattle, whereas it was 11-fold in sheep. Table 2 shows the prevalence of *Leptospira* spp. in cattle and sheep, and found 28% and 20%, respectively, as detected by PCR examination. Table-3 presents the results of the histopathological examination of 152 and 208 kidneys of cattle and sheep, respectively. Of 152 cattle kidney samples, 127 were positive for pathological lesions. Of the 152 kidneys examined, 112 (74%), 120 (79%), 126 (83%), 96 (63%), and 39 (26%) exhibited IN, glomerulonephritis, acute tubular necrosis, renal congestion, and hyaline material, respectively. Histopathological examination of the sheep kidney samples (n = 208) revealed that 178 (86%) had pathological lesions (Figures-1–3). Of the 208 kidneys examined, 145 (70%), 171 (82%), 176 (85%), 110 (53%), and 57 (27%) exhibited IN, glomerulonephritis, acute tubular necrosis, congestion, and hyaline material, respectively. In both sheep and cattle, acute tubular necrosis was the most frequent lesion recorded. The results showed no significant difference between sheep and cattle in the prevalence of kidney microscopic lesions. Similarly, no significant differences were found in the percentiles of lesions between sheep and cattle samples with the same type of lesion. Table-4 shows that all the lesions were graded independently according to their severity and pathological type. Grade + indicates that the lesions were very mild in all the pathological types recorded, grade ++ lesions were mild, whereas grade +++ lesions were moderate and grade ++++ indicates that the lesions were very severe. In the combined cattle and sheep samples, 257 out of 305 had IN, and 34%, 27%, 8%, and 5% were graded as +, ++, +++, and ++++, respectively. Among the combined cattle and sheep samples, 291 (95%) out of 305 had glomerulonephritis, with 51%, 39%, 5%, and 3% graded as +, ++, +++, and ++++, respectively. In addition, 302 (100%) out of 305 samples had acute tubular necrosis, with 27%, 51%, 19%, and 3% graded as +, ++, +++, and ++++ for ATN, respectively. Furthermore, 206 (68%) out of 305 had congestion, and 47%, 51%, 5%, and 1% were graded as +, ++, +++, and ++++, respectively. Ninety-six (31%) out of 305 samples were positive for hyaline materials, with 12%, 10%, 8%, and 2% graded as +, ++, +++, and ++++, respectively. Table-5 shows that all the lesions were independently scored according to their severity and pathological type. As shown in Table-5, we combined grades one and two (very mild + and mild ++) and grades three and four (moderate +++ and very severe ++++) to increase the number of cases in each category. Of 305 sheep and cattle samples, 257 (84%) had IN, with 71% and 13% scoring as +/++and +++/++++, respectively. Of the 305 combined samples, 291 (95%) had glomerulonephritis, of which 90% and 5% were scored as +/++ and +++/++++, respectively. In addition, 302 (100%) out of 305 patients had acute tubular necrosis, with 77% and 22% graded as +/++ and +++/++++, respectively. Furthermore, 206 (68%) out of 305 had congestion, with 62% and 6% graded as +/++ and +++/++++, respectively. Finally, 96 (31%) out of 305 were positive for hyaline materials, with 21% and 10% graded as +/++ and +++/++++, respectively. Interestingly, all kidney samples from sheep that had no pathological lesions were negative on nested PCR for the *rrs* gene of *Leptospira*.

**Table-1:** The distribution of results of kidney samples from cattle and sheep according to their type of pathological lesions, severity, and location (interstitial nephritis and acute tubular necrosis). In addition, it also shows the results of PCR investigation for Leptospirosis in cattle and sheep in Central and Northern Jordan, 2020–2021.

Severity	Cattle (%)	Leptospirosis result PCR (%)	Sheep (%)	Leptospirosis result PCR (%)
+	19 (13)	6 (4)	49 (24)	6 (3)
++	59 (39)	15 (10)	93 (45)	28 (13)
+++	37 (24)	14 (9)	28 (13)	5 (2)
++++	12 (8)	7 (5)	8 (4)	2 (1)
Total	127/152 (84)	42/152 (28)	178/208 (86)	41/208 (20)
None	25 (16)	110 (72)	30 (14)	167 (80)

PCR=Polymerase chain reaction

**Table-2:** The prevalence of *Leptospira* spp. infection in the kidneys of cattle and sheep samples collected from slaughterhouses in Central and Northern Jordan, 2020–2021.

Severity	Kidney samples collected	Leptospirosis result PCR	%
Cattle	152	42/152	28
Sheep	208	41/208	20
Total	360	83/360	24

PCR=Polymerase chain reaction

**Table-3:** The distribution of pathological lesions according to their types found in 360 kidneys of cattle and sheep from Central and Northern Jordan, 2020–2021.

Species	Sample tested (%)	Number of samples with lesions (%)	Interstitial nephritis (%)	Glomerulonephritis (%)	Acute tubular necrosis (%)	Congestion (%)	Hyaline material (%)
Cattle	152 (42)	127 (84)	112 (74)	120 (79)	126 (83)	96 (63)	39 (26)
Sheep	208 (58)	178 (86)	145 (70)	171 (82)	176 (85)	110 (53)	57 (27)
Total	360 (100)	305 (85)	257 (71)	291 (81)	302 (84)	206 (57)	96 (27)

**Table-4:** The classification of renal lesions (305 cases out of 360) according to their severity, types, and percentile in Central and Northern Jordan for Cattle and Sheep samples, 2020–2021.

Lesion severity	Interstitial nephritis (%)	Glomerulonephritis (%)	Acute tubular necrosis (%)	Congestion (%)	Hyaline material (%)
+	134 (44)	156 (51)	81 (27)	143 (47)	36 (12)
++	83 (27)	119 (39)	155 (51)	45 (15)	29 (10)
+++	24 (8)	15 (5)	58 (19)	16 (5)	24 (8)
++++	16 (5)	1 (0.3)	8 (3)	2 (1)	7 (2)
Total	257/305 (84)	291/305 (95)	302/305 (100)	206/305 (68)	96/305 (31)

**Table-5:** The classification of lesions (305 cases out of 360) into two groups of renal lesions according to their severity in Central and Northern Jordan for cattle and sheep samples.

Lesion severity	Interstitial nephritis (%)	Glomerulonephritis (%)	Acute tubular necrosis (%)	Congestion (%)	Hylein material (%)
+ ++	217 (71)	275 (90)	236 (77)	188 (62)	65 (21)
+++ ++++	40 (13)	16 (5)	66 (22)	18 (6)	31 (10
Total	257/305 (84)	291/305 (95)	302/305 (99)	206/305 (68)	96/305 (31)

### Polymerase chain reaction and phylogenetic analysis

**Figure-4:**
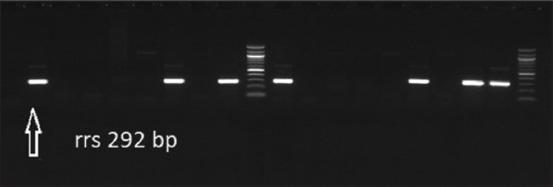
Gel electrophoresis of DNA extract of kidneys from cattle and sheep and Nested polymerase chain reaction results of the *rrs* gene at 292 bp specific for *Leptospira* spp. The four bands on the left of the figures are from cattle kidney samples while the band on the right near the ladder was from the cattle sample and was sequenced and was considered our positive control. The other two bands left to the positive control are from sheep samples.

**Figure-5:**
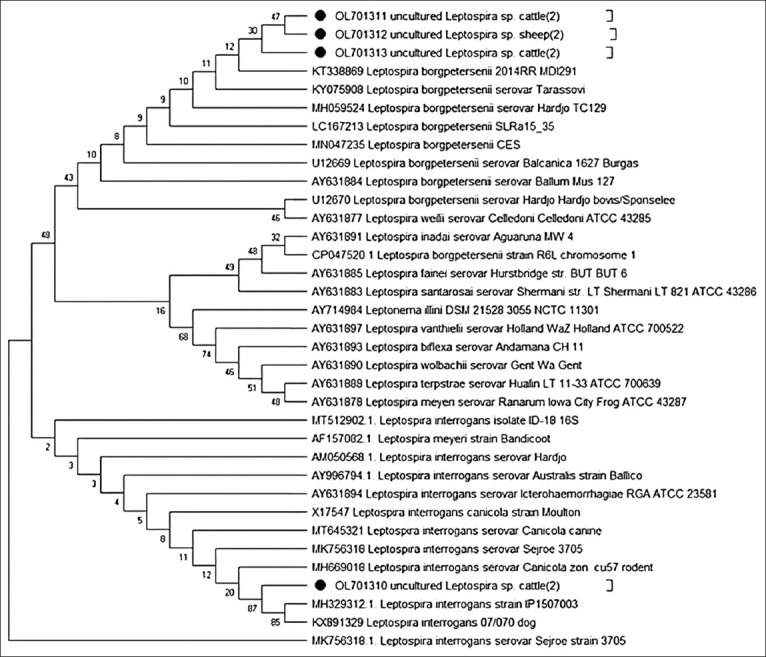
Phylogenetic analysis of partial nucleotide sequence of 16S rRNA from four uncultured *Leptospira* spp. obtained from cattle and sheep in Jordan (Black circle). The tree was reconstructed using the neighbor-joining method as implemented in MEGA-X (https://www. megasoftware.net/) The evolutionary history was inferred using the Neighbor-Joining method. The percentage of replicate trees in which the associated taxa clustered together in the bootstrap test (1000 replicates) are shown next to the branches. The tree is drawn to scale, with branch lengths in the same units as those of the evolutionary distances used to infer the phylogenetic tree. The evolutionary distances were computed using the Maximum Composite Likelihood method and are in the units of the number of base substitutions per site. This analysis involved 35 nucleotide sequences of different *Leptospira* spp. All positions containing gaps and missing data were eliminated (complete deletion option).

*Leptospira* spp. isolates from uncultured kidney specimens collected from cattle and sheep were analyzed by PCR. *Leptospira* isolates with a 292-bp product size that was positive for 16S rRNA were detected in 23.1% of all samples (n = 360), specifically in 28% of sheep, and 20% of cattle kidney samples. The prevalence of samples that were positive for *Leptospira* spp. did not vary significantly between the two species (p < 0.078) (Figure-4). Four samples (two ovine and two bovine) were sequenced and deposited in GenBank under accession numbers from OL701310 to OL701313. A BLAST search on the NCBI confirmed the presence of *Leptospira* spp. 16S rRNA sequences in these samples. Nucleotide similarities of the obtained 16S rRNA *Leptospira* spp. sequences were high between OL701311 and OL701313, ranging from 94.7% to 99.6%, whereas that of OL701310 was lower (91.4%–93.6%). Furthermore, the BLAST search and nucleotide similarities for OL701311 to OL701313 had the highest similarities with different *Leptospira borgpetersenii* strains, whereas OL701310 had the highest nucleotide similarity (99.2%) with *Leptospira interrogans* strain IP1507003 (accession number: MH329312.1) (Table-6). These isolates were obtained from naturally infected cattle from Uruguay. Furthermore, this *Leptospira* strain is pathogenic and is considered a significant risk factor for human leptospirosis in Uruguay [17]. Similarly, phylogenetic analysis revealed that OL701311–OL701313 clustered together with different serovars of *L*. *borgpetersenii*, whereas OL701310 clustered with the *L. interrogans* clade (Figure-5).

**Table-6:** Estimates of evolutionary divergence between *Leptospira* 16S ribosomal RNA gene (*rrs*) sequences.

*Leptospira* strains	OL701310	OL701311	OL7013112	OL701313
OL701310_uncultured_*Leptospira* _spp._cattle (2)	100.0	93.6	92.7	91.4
OL701311_uncultured_*Leptospira* _spp._cattle (2)	93.6	100.0	99.6	97.6
OL701312_uncultured_*Leptospira* _spp._sheep (2)	92.7	99.6	100.0	94.1
OL701313_uncultured_*Leptospira* _spp._cattle (2)	91.4	97.6	94.1	100.0
MH329312.1._*Leptospira* _*interrogans*_strain_IP1507003	99.2	93.1	92.6	93.6
MK756318.1._*Leptospira* _*interrogans*_serovar_Sejroe_strain_3705	98.8	94.6	94.1	95.1
MT512902.1._*Leptospira* _*interrogans*_isolate_ID-18_16S	98.8	94.6	94.1	95.1
AF157082.1._*Leptospira* _meyeri_strain_Bandicoot	98.8	94.6	94.1	95.1
AM050568.1._*Leptospira* _*interrogans*_serovar_Hardjo	98.8	95.0	94.6	95.5
AY631884_*Leptospira* _*borgpetersenii*_serovar_Ballum_Mus_127	91.3	96.8	93.1	95.8
U12670_*Leptospira* _*borgpetersenii*_serovar_Hardjo_Hardjo_bovis/Sponselee	90.2	95.8	92.1	94.8
U12669_*Leptospira* _*borgpetersenii*_serovar_Balcanica_1627_Burgas	91.3	96.8	93.1	95.7
AY631894_*Leptospira* _*interrogans*_serovar_Icterohaemorrhagiae_RGA_ATCC_23581	94.4	93.7	89.9	92.7
X17547_*Leptospira* _*interrogans*_canicola_strain_Moulton	94.9	93.2	89.9	92.3
AY631883_*Leptospira* _santarosai_serovar_Shermani_str._LT_Shermani_LT_821_ATCC_43286	90.2	93.5	89.6	92.6
AY631877_*Leptospira* _weilii_serovar_Celledoni_Celledoni_ATCC_43285	91.2	95.4	91.7	94.5
MN047235_*Leptospira* _*borgpetersenii*_CES	91.3	96.8	93.1	95.8
LC167213_*Leptospira* _*borgpetersenii*_SLRa15_35	91.3	96.8	93.1	95.8
MH059524_*Leptospira* _*borgpetersenii*_serovar_Hardjo_TC129	91.3	96.8	93.1	95.8
KY075908_*Leptospira* _*borgpetersenii*_serovar_Tarassovi	91.3	96.8	93.1	95.8
KT338869_*Leptospira* _*borgpetersenii*_2014RR_MDI291	91.3	96.8	93.1	95.8
KX891329_*Leptospira* _*interrogans*_07/070_dog	95.7	92.6	89.8	91.7
MT645321_*Leptospira* _*interrogans*_serovar_Canicola_canine	94.9	93.2	89.4	92.3
MK756318_*Leptospira* _*interrogans*_serovar_Sejroe_3705	95.3	93.2	89.3	91.4
MH669018_*Leptospira* _*interrogans*_serovar_Canicola_zon._cu57_rodent	94.9	93.2	89.4	92.3

## Discussion

This study investigated the role of food-producing animals in the epidemiology of leptospirosis in Jordan. Kidney disorders in ruminants are caused by multiple infectious agents, such as *Bacillus*, *Escherichia coli*, *Leptospira*, *Klebsiella*, *Staphylococcus*, *Streptococcus*, *Corynebacterium* spp., *Pseudomonas aeruginosa*, *Salmonella*, and *Proteus*, all of which are found worldwide, including in the Middle East [11]. These pathogens cause serious renal problems and economic losses. This study focused on *Leptospira* spp. due to their zoonotic potential. Furthermore, *Leptospira* spp. is a diverse group of pathogenic bacteria (including at least nine genospecies and over 200 serovars), which annually leads to more than 1.03 million infections and 50,000 deaths in humans due to leptospirosis [4]. *Leptospira* is also considered a multiorgan disease that can cause kidney infections (such as tubulointerstitial nephritis, tubular dysfunction, and acute renal failure) and reproductive organ problems (e.g., abortion) [18]. Polymerase chain reaction is a rapid method that can provide fast laboratory diagnosis and is highly specific and sensitive for identifying the causative agent of infections [19]. In addition, PCR is a good diagnostic technique in cases with negative culture or serological results due to its high sensitivity and fast turnaround time. In general, the variations between the prevalence of *Leptospira* isolates in our study and those reported in other studies have many reasons other than true prevalence. These reasons include differences in the sensitivity of PCR tests due to different primers used and sample types evaluated, in addition to differences in host and pathogen genetic factors. Polymerase chain reaction analysis showed that 23.1% of the renal tissues were positive for *Leptospira* spp. (28% in cattle and 20% in sheep). A recent study in Iran that analyzed cattle renal samples using nested PCR found that 40.8% of the samples were positive for *Leptospira* [20]. In another study, PCR analysis of blood samples revealed that 21% of cattle and 17.5% of sheep samples were positive for *Leptospira* spp. [21]. In a study conducted in southwest Iran [22], PCR analysis of cattle kidney samples revealed a higher prevalence (79.2%) than our results (28%). In our study, 20.5% of combined kidney samples had histopathological lesions, 7.5% had chronic nephritis, and 7.3% had white spots. In our study, the prevalence of *Leptospira* spp. in the kidneys of cattle was 28%. In comparison, this was lower than the prevalence rates reported in two studies in Iran (40.8% and 79%), although a third study reported a lower prevalence (21%). Our study found that, 19.7% of sheep kidney tissue tested by PCR were positive for *Leptospira* spp., which is similar to another study conducted in Iran [22]. A study in New Zealand performed quantitative PCR (qPCR) analysis of kidney tissues and found that the prevalence of *Leptospira* spp. was 21% in cattle and 29% in sheep [23]. These findings are interesting in comparison to those of our study, at 28% and 20% for cattle and sheep, respectively. A study in South Africa reported a 27% prevalence of *Leptospira* spp. in cattle kidney samples using PCR but did not observe any renal lesions [24]. In Brazil, sheep kidney tissues were analyzed using PCR, histopathological examination, and immunohistochemistry (IHC), and 4.1% of the samples were verified for *Leptospira* spp. DNA, whereas IHC revealed that 62.5% of the samples were positive for *Leptospira* spp. antigen. The IHC results were consistent with histological changes compatible with leptospirosis reported previously [25]. In Chile, 11.5% of urine samples collected from cattle with clinical signs of leptospirosis were positive for leptospirosis by qPCR [26]. Furthermore, another study in Brazil, conducted on cattle with reproductive problems, found that 21.6% of the urine samples collected were positive for *Leptospira* spp. [27]. Similarly, a study in Iran found a 14% prevalence of *Leptospira* in stomach content samples from aborted bovine fetuses [28]. Most published studies on small and large ruminant renal diseases have used serological, culture, pathological, and molecular diagnostic techniques, either separately or in combination, to assess the presence of *Leptospira* spp. [29, 30]. Few reports have described the use of histopathological, culture, serological, and PCR tests to assess renal diseases. Discrepancies in the results and analyses across laboratories worldwide are critical issues to consider, as they use different combinations of diagnostic tests and different types of tissue samples. The limitations of obtaining proper tissue samples at the appropriate time make comprehensive research difficult but useful. Based on nucleotide similarity and the phylogenetic tree, our screening allowed us to identify two pathogenic *Leptospira* strains: *L. interrogans* (OL701310) and *L. borgpetersenii* (OL701311 to OL701313). However, we cannot exclude the possibility that we missed identifying positive samples due to the restriction of sample collection to slaughterhouses and the quality of the sequencing results. The 16S rRNA sequences of the identified *Leptospira* strains were related to the lineages involved in human diseases, suggesting that food-producing animals (cattle and sheep) may play significant epidemiological and pathological roles in the incidence of leptospirosis in animals and humans in Jordan [17]. Considering that cattle and sheep are species widely distributed in Jordan, the epidemiology and impact of leptospirosis in animals and humans in Jordan require greater consideration. The previous report has indicated that rodents may be the maintenance hosts of *L. interrogans* and *L*. *kirschneri* [31]. Direct and indirect contact with infected rodents is a well-documented risk factor for acquiring leptospirosis in humans and animals [32].

Histopathological findings revealed multifocal infiltration of mononuclear cells, including lymphocytes and a few plasma cells, in the renal interstitial tissues affected by “white spotted kidneys” associated with leptospirosis [22].The histopathological lesions identified in our study included IN, tubular necrosis, and cortical and medullary inflammation (lymphocytic and macrophagic). Other renal samples were test-negative or considered non-infectious based on the criteria proposed in the previous reports [11, 33–35]. In Jordan, no reports have described the pathological, bacteriological, or molecular investigation of the kidneys of cattle or sheep. Therefore, this study is the first to emphasize pathological changes and PCR analysis for the presence of *Leptospira*. This study investigated 360 kidneys of apparently healthy cattle (152) and sheep (208) collected from slaughterhouses in Central and Northern Jordan. Histopathological examination of 152 sheep kidney samples revealed that 127 (83%) had pathological lesions. Our study findings differ from those previously reported in that we classified the histopathological lesions into four categories and graded their severity from + to ++++. Comparing our findings with those previously reported, we found that those from previous reports were lower than our results when all the categories were included in the study. For example, a histopathological study on the kidneys of apparently healthy sheep in Iraq found that 38% had microscopic lesions [13], whereas, in our study, we found that 86% of the sheep samples and 84% of the cattle samples had microscopic lesions. However, if we limit our comparison to the +++ and ++++ groups, which showed clear lesions, the prevalence of IN and glomerulonephritis in the cattle samples was 20% and 9%, respectively, whereas in the sheep samples, it was 8% and 2%, respectively. The prevalence rates in the literature vary widely. El-Mashad *et al*. [10] found that 17.4% of cattle kidney samples collected from a Cairo slaughterhouse had IN, compared to 28.5% in sheep. For glomerulonephritis, the prevalence is higher in our study than that in the El-Mashad *et al*. [10] study, at 22% and 26% of the cattle and sheep samples, respectively. A study in Iran reported that 165 (55%) out of 300 condemned bovine kidneys had IN (white-spotted kidneys), but only one kidney sample had glomerulonephritis [35]. Furthermore, they reported that 15 (5%) out of 300 kidney samples had no lesions. In comparison, our study reported 25 (16.4%) out of 152 kidneys without lesions. Another study in Iran showed that IN was found in 24 (13%) of the 180 bovine kidneys examined and was associated with leptospirosis, whereas 19 out of the 24 were positive for *Leptospira* on PCR. A study in Mosul, Iraq, found that IN was the most common histopathological finding in calves (20%) by gross examination [36]. A large survey in Ghana from 2002 to 2014 found that 33% of the condemned kidneys in cattle were diagnosed with IN, suggesting significant economic losses [37]. Furthermore, a study conducted in Australia on the kidneys of cattle found that gross and histopathological lesions were consistent with focal chronic IN. Interestingly, based on the results of a microscopic agglutination test, they suggested that neither *Leptospira* spp. nor active infection by other bacteria was associated with a “white-spotted kidney” [38]. In contrast, a PCR-based diagnosis of the *LipL32* gene revealed an association between a white-spotted kidney and leptospirosis [22].

## Conclusion

Our findings show that the prevalence of *Leptospira* spp. was 20% (41/208) in sheep and 28% (42/152) in cattle kidney samples. The most common pathological lesion found in kidney samples was acute tubular necrosis, followed by IN. Four samples were sequenced and deposited in the GenBank database. The NCBI BLAST search further confirmed that the kidney samples contained the *Leptospira* spp. The nucleotide similarities of the three *Leptospira* spp. sequences obtained, OL701311 to OL701313, and *L. borgpetersenii* strains were very high, whereas OL701310 had the highest nucleotide similarity (99.2%) with *L. interrogans* strain IP1507003 (accession number: MH329312.1), which was isolated from naturally infected cattle in Uruguay. Phylogenetic analysis revealed that OL701311-OL701313 clustered with different serovars of *L. borgpetersenii*, whereas OL701310 clustered with the *L. interrogan*s clade.

## Authors’ Contributions

NQH and MHG: Conceptualized, designed, and planned the study, as well as drafted the proposal and manuscript. ZAM: Sampling, conducted the study, analyzed and visualized the results. IMA: Analysis of PCR and sequencing results. All authors have read, reviewed, and approved the final manuscript.
